# Assessment of In Vitro Immunostimulatory Activity of an Adjuvanted Whole-Cell Inactivated *Neisseria gonorrhoeae* Microparticle Vaccine Formulation

**DOI:** 10.3390/vaccines10070983

**Published:** 2022-06-21

**Authors:** Priyal Bagwe, Lotika Bajaj, Rikhav P. Gala, Martin J. D‘Souza, Susu M. Zughaier

**Affiliations:** 1 Vaccine Nanotechnology Laboratory, Center for Drug Delivery Research, College of Pharmacy, Mercer University, Atlanta, GA 30341, USA; priyal.bagwe@live.mercer.edu (P.B.); lotika.bajaj@live.mercer.edu (L.B.); dsouza_mj@mercer.edu (M.J.D.); 2 Fraunhofer USA, Center Mid-Atlantic, Biotechnology Division, 9, Innovation Way, Newark, DE 19011, USA; rikhav.praful.gala@gmail.com; 3 Department of Basic Medical Sciences, College of Medicine, QU Health, Qatar University, Doha P.O. Box 2731, Qatar

**Keywords:** *Neisseria gonorrhoeae*, microparticles, vaccines, antigen presentation, autophagy, gonorrhea

## Abstract

The emergence of drug-resistant gonorrhea infections worldwide combined with the lack of a vaccine is alarming. We prepared a novel microparticulate (MP) vaccine formulation using whole-cell inactivated *Neisseria gonorrhoeae* as the vaccine antigen, with Alum and AddaVax™ as vaccine adjuvants. The adjuvanted vaccine MP formulation was assessed for in vitro immunostimulatory activity, autophagy, and antigen presentation ability. The data shows that the adjuvanted gonococci vaccine MP enhanced autophagy induction in antigen presenting cells (APCs) compared to gonococci vaccine MP without adjuvants, which is important for enhancing antigen presentation. In addition, the adjuvanted vaccine formulation increased the surface expression of antigen presenting molecules MHCI and MHCII as well as co-stimulatory molecules CD40 and CD86 on the surface of dendritic cells. In addition, the gonococci vaccine microparticles at lower doses did not significantly increase the expression of the death receptor CD95 in APCs, which when elevated leads to suboptimal antigen presentation and reduced immune responses. The adjuvanted whole-cell inactivated gonococci microparticle vaccine formulation enhanced antigen uptake, processing, and antigen presentation.

## 1. Introduction

According to the World Health Organization (WHO), about one million new cases of sexually transmitted infections occur every day worldwide [1]. Gonorrhea is the second most reported bacterial infection in the United States [2]. Gonorrhea infection occurs in around 100 million people throughout the world per year [2,3,4]. *Neisseria gonorrhoeae,* a gram-negative diplococcus, is the primary causative pathogen. It is a strict human pathogen transmitted through sexual contact or perinatally from mother to newborn during childbirth [5]. *N. gonorrhoeae* is rapidly developing resistance to the antibiotics used for its treatment. The rise in drug-resistant gonorrhea infections worldwide is alarming. Today, the United States has just one recommended treatment option against drug-resistant gonorrhea. Ceftriaxone is the last available treatment option [6]. The occurrence of gonorrhea infection is more common in Africa and other developing nations [5]. Untreated gonorrhea infection in women may lead to pelvic inflammatory disease, increased risk of ectopic pregnancy and infertility, and potentially increases a person’s risk of acquiring or transmitting the human immunodeficiency virus (HIV) infection [7]. With the emergence of antibiotic-resistant gonococcal strains, there is a need for vaccine. There is no approved vaccine for gonorrhea in the market, despite the vast incidence of this disease [8]. The Centers for Disease Control and Prevention (CDC) and the WHO has listed gonorrhea vaccine development research as a high priority [9,10,11].

Gonorrhea vaccine development has been challenging due to the antigenic variability of *N. gonorrhoeae* and the ability of this pathogen to inhibit the development of protective adaptive immune responses [9]. Previous attempts to develop gonorrhea vaccine candidates such as whole cell attenuated or subunit recombinant vaccines were unsuccessful [12,13,14,15]. The current gonorrhea vaccine candidates include gonococcal outer membrane vesicles (OMV), lipopolysaccharide epitope, and subunit vaccines (protein-based, purified) [8]. Moreover, *N. meningitidis* and *N. gonorrhoeae* share most of their genetic sequence [16,17]. Thus, researchers are currently investigating the ability of meningococcal OMVs to induce cross protective anti-gonococcal antibodies [18,19,20]. A search in clinicaltrials.gov (on 2 January 2022) with keywords ‘gonorrhea vaccine’ revealed six studies [19,21,22,23,24,25]. One Phase 4 clinical trial with 11 participants is completed and has results. The participants received the group B meningitis vaccine (Bexsero™). Another five studies also investigate meningococcal OMV-based vaccines’ efficacy against gonorrhea infection. Advanced strategies are needed to control the global spread of gonococcal disease. The global roadmap to advance vaccine development against sexually transmitted infections has catalyzed the gonorrhea vaccine development process [10].

Vaccines are the most effective public health approach to reduce the burden of infectious diseases. Upon vaccination, the innate immune system is activated. Antigen presentation is the connecting link between innate and adaptive arms of the immune system. Antigen presenting cells (APCs) such as dendritic cells (DCs) and macrophages take up the antigen, process it, and then present it to the naïve T cells to initiate the immune response cascade. Vaccine antigens are loaded into the major histocompatibility complexes (MHC) molecules for antigen presentation, which is a critical step required for T cell activation. MHCI molecules are recognized by the cytotoxic- CD8+ T cells (expressed on all cells), and MHCII molecules are recognized by the helper- CD4+ T cells (expressed on APCs) [26,27,28,29].

We have developed a whole-cell inactivated vaccine of *N. gonorrhoeae* encapsulated into a biodegradable polymer matrix as a microparticle (MP) formulation. Previous studies in our laboratory have shown immune response in mice vaccinated using this whole-cell gonococci vaccine strategy [30]. We have demonstrated a potentially functional vaccine candidate which generated antigen-specific CD4 and CD8 T lymphocyte responses in a pilot study. However, those vaccine particles were not adjuvanted. In this study, we assessed the immune stimulatory activity of this novel gonorrhea vaccine formulated with adjuvants and investigated whether it has the potential to enhance antigen presentation. To improve the antigenicity of the particulate gonorrhea vaccine MPs, various adjuvants were screened for their potential to enhance the antigen presentation by dendritic cells (DCs). We formulated MP containing Alum (Alhydrogel^®^) and AddaVax™ (Analog of MF59) as vaccine adjuvants. Alum is a widely used FDA-approved vaccine adjuvant. Aluminum-based adjuvants enhance immunogenicity by producing a depository at the site of administration, leading to localized inflammation, which facilitates the recruitment of immune cells and consequently enhances antigen uptake and presentation. This slow and sustained release mechanism allows for an increased interface between the antigen and immune cells [31,32]. AddaVax™ is a squalene-based nano-emulsion formulation (oil in water) equivalent to MF59^®^ [33]. We used AddaVax^™^ in our adjuvant MP formulation as its equivalent to MF59 which is used in the adjuvanted FLUAD flu vaccine approved in the US for people 65 years of age or older [34]. MF59^®^ prompts robust T cell responses, and it is a potent adjuvant that exerts vaccine-specific antibody and B cell responses [35]. We have previously reported that AddaVax™ (Analog of MF59) and Alum (Alhydrogel^®^) are superior to toll-like receptor (TLR)-based adjuvants in enhancing DC maturation and antigen presentation on the MHC, followed by co-stimulatory molecule expression in DCs induced with meningococcal capsular polysaccharides vaccine microparticle formulation [31,36].

Here, we evaluated the in vitro antigen presentation of the novel gonococci vaccine formulation consisting of whole-cell inactivated *N. gonorrhoeae* in MP adjuvanted with Alum MP and AddaVax™ MP.

## 2. Materials and Methods

### 2.1. Materials

*Neisseria gonorrhoeae* strain CDC-F62 was provided by Dr. William M. Shafer (Emory University, Atlanta, GA, USA). The isolates were stored at −80 °C until further use. The adjuvants, Alhydrogel^®^ (Alum) and AddaVax™ (Analog of MF59), were purchased from Invivogen (San Diego, CA, USA). Bovine serum albumin (BSA) was purchased from Fisher Scientific (Waltham, MA, USA). The protein content analysis was carried out using the Micro BCA™ Protein Assay Kit purchased from Thermo Fischer Scientific (Rockford, IL, USA). Sulfanilamide, N-1-naphthyl ethylenediamine dihydrochloride (NED), and sodium nitrite were purchased from Thermo Fisher Scientific (Rockford, IL, USA). 3-(4,5-dimethylthiazol-2-yl)-2,5-diphenyl tetrazolium bromide (MTT) was purchased from Sigma Aldrich (St. Louis, MO, USA). Dendritic cells (DC 2.4) were purchased from American Type Culture Collection (ATCC) (Manassas, VA, USA). Antibodies used to stain cells for the purpose of flow cytometry analysis [Phycoerythrin (PE) bound MHC I (AF6-88.5.5.3), Fluorescein isothiocyanate (FITC) bound MHC II (M5/114.15.2), PE anti-mouse CD40 (3/23), FITC anti-mouse CD80 (16-10A1), and PE anti-mouse CD95 (SA367H8)] were purchased from eBiosciences (San Diego, CA, USA) and Biolegend (San Diego, CA, USA). RAW 264.7 macrophage cell line was stably transfected with a construct encoding the autophagy marker GFP-LC3 (a generous gift from Dr. Alfred Merrill, Georgia Institute of Technology, Atlanta, GA, USA).

### 2.2. Methods

#### 2.2.1. Preparation of Whole-Cell Inactivated *N. gonorrhoeae* Vaccine Antigen

*N. gonorrhoeae* strain CDC-F62 was used as the basis for this vaccine as previously described [30]. It was grown from freezer stock on GC agar containing defined supplements under 5% CO_2_ at 37 °C. Pileated colonies were selected and further sub-cultured on a GC agar plate overnight. The fresh growth of pileated colonies was used to inoculate two flasks containing 300 mL/flask of GC broth containing defined Supplement I and II with sodium bicarbonate in a 1L flask. The OD600 was ~0.2 at the start of bulk culture. The flasks were incubated with shaking at 37 °C till the late mid-log phase, i.e., when OD600 reached ~0.5. The growth of gonococci was stopped by adding 10% formalin (*v*/*v*) and left to gentle shaking overnight at room temperature. The formalin-fixed bacterial pellets were then harvested. The centrifugation was carried out at 5000× *g* for 15 min at 4 °C. The harvested pellets were washed three times with sterile phosphate buffer saline (PBS) and centrifuged as above. The final collected pellets were pooled and vortexed thoroughly and saved as a very dense suspension at −80 °C. The formalin-fixed whole-cell inactivated *N. gonorrhoeae* was examined using a scanning electron microscope (Phenom-World Pure Scanning electron microscope, Phoenix, AZ, USA) using accelerated voltage of 20 kV at 7500×. Whole-cell *N. gonorrhoeae* was quantified for protein content before encapsulation using Micro BCA™ Protein Assay Reagent Kit (Thermo Fischer Scientific, Rockford, IL, USA).

#### 2.2.2. Gonococci Vaccine Microparticles and Adjuvant Microparticles Formulation

Gonococci vaccine microparticles (Gc-MP) and adjuvant MP (Alum and AddaVax™) were prepared by a method previously developed in our laboratory using the Buchi Mini Spray Dryer B-290 [30]. Briefly, pre cross-linked BSA solution was made using glutaraldehyde (25% aqueous solution purchased from Fischer Scientific, Pittsburgh, PA, USA) at a concentration of 200 µL for every 1 g of BSA. This solution was kept on a magnetic stirrer for stirring at 300 RPM overnight in a dark at room temperature. Excess glutaraldehyde was neutralized with sodium bisulfite (10 mg) the next day [37]. We prepared 100 mg of vaccine MP with 10% antigen loading, for which 10 mg of the formalin-fixed whole-cell of *N. gonorrhoeae* (4 mL of 2.5 mg/mL stock solution) and 90 mg of pre-cross-linked BSA were mixed. Similarly, we prepared 100 mg of Alum and 100 mg of AddaVax™ MP with 10% loading each. The resulting formulations were sprayed separately through a 0.5 mm nozzle (nozzle temperature of −5 °C); the atomizing gas was compressed air. Inlet temperature for the spray-drying process was 120 °C with the aspirator at 100%, and a feed-flow rate of 20 mL/h was used to obtain the *N. gonorrhoeae* vaccine MP. The particle size of the MP was measured using dynamic light scattering (DLS), and surface charge (zeta potential) was measured using a zeta sizer (Malvern Zetasizer Nano ZS, Westborough, MA, USA).

#### 2.2.3. Autophagy Induction Assessment

To investigate the impact of the gonococci vaccine MP on autophagy induction, macrophage cells (RAW264.7) were stably transfected with the autophagy marker GFP-LC3 [38]. Briefly, GFP-LC3 RAW264.7 macrophages were harvested and adjusted to 1 × 10^6^ cells/mL. These cells were seeded on glass coverslips (Surgipath Medical Industries Inc., Richmond, IL, USA), placed in tissue culture plates, and incubated overnight. The adherent cells were washed and placed in Dulbecco’s Modified Eagle Medium (DMEM) (with 10% heat-inactivated Fetal Bovine Serum (FBS)) before stimulation with vaccine MP and incubated at 37 °C overnight with 5% CO_2_. The cells were exposed to different treatment groups, including cells only (negative control—no treatment), Gc-MP (high, medium, and low concentrations; Gc-MP- 1 mg/mL stock solution in PBS, 100 µg/mL for high, 50 µg/mL for medium and 25 µg/mL for low doses), Gc-MP+ Alum MP + AddaVax™ MP, and only adjuvants (Alum MP, AddaVax™ MP; 50 µg/mL) overnight. Initial screening of the adjuvants was performed to assess the activity singularly. These stimulated cells were then washed thrice with PBS and fixed with paraformaldehyde (4% *w*/*v*). After fixation, they were stained with (4′,6-diamidino-2-phenylindole) DAPI nuclear (Thermo Fischer Scientific, Rockford, IL, USA) for 5 min. The inverted coverslips were sealed by mounting them on glass slides. Autophagy was monitored using confocal microscopy (Olympus IX8S1F-3, Olympus Corporation, Tokyo, Japan). We observed multiple fields in each glass coverslip, captured images, and analyzed using FV10-ASW software. Autophagy induction index was systematically calculated based on the fluorescence intensity of GFP-LC3 reflecting autophagic vacuoles and DAPI staining of nuclei as previously detailed [39].

#### 2.2.4. Nitric Oxide Release Measurement by the Griess Reaction

In vitro immunostimulatory activity of gonococci vaccine, MP with and without adjuvant MP was evaluated using the spectroscopic Griess assay in murine macrophage (RAW 264.7). Briefly, RAW 264.7 macrophages were plated in a 48 well plate at a seeding density of 2.5 × 10^4^ cells/well. The cells were exposed to different treatment groups, including Gonorrhea vaccine microparticles (Gc-MP-High) 100 µg/mL, Gc-MP-Medium 50 µg/mL, Gc-MP-Low 25 µg/mL, Gc-MP with Alum microparticles (Gc-MP+ Alum MP) 50 µg/mL of each, Gc-MP with AddaVax™ microparticles (Gc-MP+ AddaVax™ MP) 50 µg/mL of each, unstimulated control cells, Alum MP, and AddaVax™ MP alone for 24 h. Initial screening of the adjuvants was done to assess the activity singularly. Culture supernatants were collected and treated with the Griess reagent system (sulfanilamide and NED) for the detection of nitrite accumulation by the formation of a pink-colored azo compound. The pink color intensity was measured at 540 nm to calculate the concentration of nitrite. The known concentrations of sodium nitrite were used as standards as previously described [40].

#### 2.2.5. In Vitro Cytotoxicity Evaluation Using the MTT Method

In vitro safety of vaccine, MP was evaluated by 3-(4,5-dimethylthiazol-2-yl)-2,5-diphenyl tetrazolium bromide (MTT) cell viability assay. In this assay, viable cells convert yellow-colored MTT reagent into purple-colored formazan through an enzymatic pathway. The intensity of the purple dye is directly proportional to the viability of cells and is measured at 570 nm. Murine dendritic cells (DC 2.4) were plated in a 48 well plate at a seeding density of 2.5 × 10^4^ cells/well. The cells were exposed to different treatment groups, including cells only (control—no treatment), DMSO treated cells (control), and various concentrations of adjuvanted vaccine MPs. After the exposure period, cells were washed to remove extracellular particles and incubated with MTT reagent for 2 h to form the formazan. The purple-colored formazan precipitate was dissolved, and the absorbance was measured at 570 nm.

#### 2.2.6. Dendritic Cell Uptake Study

The uptake of fluorescence isothiocyanate (FITC)- labelled blank BSA microparticles by murine dendritic cells (DC 2.4) was evaluated by fluorescence microscopy. The dye loaded MPs were prepared using the same method as discussed above. The dendritic cells (DC 2.4) were plated in a 24-well plate at a density of 5 × 10^4^ cells per well. The cells were induced with 100 µg of FITC-labelled microparticles and incubated at 37 °C for 2 h. The adherent cells were then washed to remove the microparticles not up taken. The cells were then observed using a fluorescence microscope (Lionheart FX Automated Microscope, Agilent Technologies, Santa Clara, CA, USA).

#### 2.2.7. Quantification of MHCI, MHCII, CD40 and CD86 Surface Expression on Dendritic Cells

Dendritic cells (DC 2.4) were plated at a density of 5 × 10^4^ cells per well on a 24 well plate and incubated at 37 °C for 24 h to adhere and stabilize. The adherent cells were induced with 100 µg of *N. gonorrhoeae* loaded microparticles along with the equal quantity of adjuvant MP (Alum MP, AddaVax™ MP) in each well and incubated at 37 °C for 16 h. Cells were also induced with adjuvant particles only. Based on the initial assessment, we decided selection of individual adjuvant MPs, both Alum and AddaVax MPs were carried forward. The exposed cells were then washed to remove extracellular particles before staining with allophycocyanin (APC) and FITC labeled MHCI and MHCII markers, respectively (eBioscience laboratories, San Diego, CA, USA). The stained cells were then washed, and fluorescence intensity was quantified using the BD Accuri C6 flow cytometer (Becton, Dickinson and Company, San Diego, CA, USA). The same procedure and controls were used for evaluating the expression of co-stimulatory molecules, CD40 and 86 in DCs (Biolegend, San Diego, CA, USA).

#### 2.2.8. Evaluation of Surface Expression of Death Receptor CD95 on Dendritic Cells

The expression of CD95 was examined by plating 5 × 10^4^ dendritic cells (DC 2.4) per well in a 24 well plate and incubated at 37 °C for 24 h to adhere and stabilize. Adherent cells were induced with 10, 25, 50, and 100 μg per well of gonorrhea vaccine MP (with and without adjuvants) and further incubated at 37 °C overnight. A similar concentration of fixed whole-cell gonococci suspension and equal amounts of blank MP were used as controls. The induced cells were then washed to remove excess MP not taken up by DCs and stained for the CD95 marker. Trypan blue exclusion assay was used to measure the viability of APCs, as described before [32]. Briefly, the harvested cells were treated with FITC-labeled CD95 marker (eBioscience laboratories, San Diego, CA, USA) for 1 h at 4 °C. The cells were washed, and sample measurements were then acquired on the BD Accuri C6 flow cytometer (Becton, Dickinson and Company, San Diego, CA, USA).

#### 2.2.9. Statistical Analysis

All the experiments were repeated as three independent experiments with three replicates in each experiment, unless otherwise stated. For statistical analysis, if the data was normally distributed then Ordinary one-way ANOVA or Brown-Forsythe & Welch ANOVA tests were used for independent groups and Two-way ANOVA was used for dependent groups. If the data was not normally distributed then, Kruskal-Wallis test was used. A post-hoc Tukey test was used to compare between means and post-hoc Dunnett test was used to compare means to control. Mean values ± standard deviation and *p*-values were determined individually for all experiments with GraphPad prism 8.4.3 software (GraphPad Software, San Diego, CA, USA).

## 3. Results

### 3.1. Characterization of the Microparticles

The protein content of the formalin-inactivated whole-cell Gc before encapsulation was found to be 2.5 mg/mL (Table 1). The MP were found to be spherical in shape, and the percent yield after the process of spray drying was found to be approximately 85% *w*/*w* (Table 1). Loss in the yield of MP can be attributed to sticking of particles to the spray dryer cylinder and cyclone separator. The particles size was measured by dynamic light scattering. The high negative zeta potential indicated the high stability of MP. The polydispersity index indicated the uniform distribution of particles and that there would be no agglomeration of the particles when reconstituted in media (Table 1).

### 3.2. Adjuvanted Gonococci Vaccine Microparticles Enhanced Autophagy Induction in Antigen Presenting Cells

**Figure 1:**
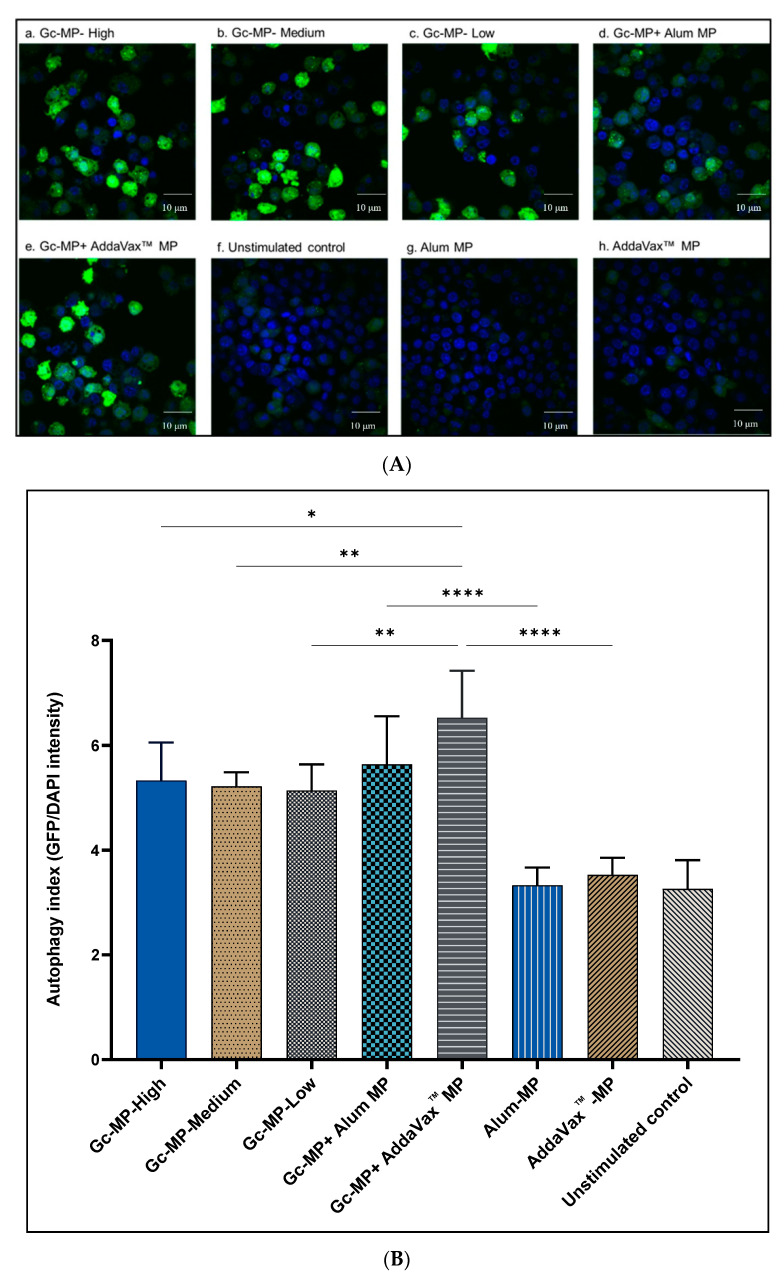
Autophagy induction in GFP-LC3-RAW264.7 macrophages induced with gonococci vaccine microparticles (Gc-MP) with or without adjuvant MP. Macrophages were adjusted to 1 × 10^6^ cells/mL and induced with various doses of vaccine microparticles for 18 h. Gonorrhea vaccine microparticles Gc-MP-High 100 µg/mL, Gc-MP-Medium 50 µg/mL, Gc-MP-Low 25 µg/mL, Gc-MP with Alum microparticles (Gc-MP+ Alum MP) 50 µg/mL of each, Gc-MP with AddaVax™ microparticles (Gc-MP+ AddaVax™ MP) 50 µg/mL of each. Unstimulated control, Alum MP, and AddaVax™ MP alone were used. (**A**): Representative confocal microscopy images of autophagy induction in GFP-LC3-RAW264.7 macrophages exposed to different treatments. Green fluorescence protein-conjugated light chain 3 (GFP-LC3 puncta) indicates active autophagy and DAPI (blue) reflects nucleus staining. (**B**): Autophagy index quantitation was calculated by dividing GFP intensity over DAPI intensity using systemic image analysis of at least 6 different images for each treatment. All treatments were repeated six times. Data are expressed as mean ± SD, Brown-Forsythe, and Welch ANOVA test. * *p* < 0.05 significant, ** *p* < 0.01 very significant, **** *p* < 0.001 extremely significant.

Here we investigated the effect of adjuvants on autophagy induction by gonococci vaccine microparticles (Gc-MP) adjuvanted with Alum (Gc-MP+ Alum MP) or AddaVax™ MP in APCs. To this end, murine RAW264.7 macrophages stably transfected with GFP-tagged LC3 construct, the marker of autophagy, were employed. Autophagy induction was monitored by measuring the fluorescence intensity of the LC3 puncta using confocal microscopy. GFP-LC3 puncta reflects formation of autophagic vacuoles in the cytoplasm which as previously demonstrated correlates with active autophagy flux in these immune cells [39,41,42]. The autophagy index was calculated using image analysis software FV10-ASW as previously described [39]. The results indicate that gonococci MP combined with adjuvant MP (Gc-MP+ Alum MP and Gc-MP+ AddaVax™ MP) induced significantly (*p* < 0.001) more autophagy in macrophages than adjuvants MP alone or Gc-MP alone (Figure 1A,B). However, unstimulated macrophages showed minimal autophagy induction consistent with the basal levels during cellular homeostasis (Figure 1A,B).

### 3.3. Antigen Presentation Assessment in Antigen Presenting Cells Exposed to Gonococci Vaccine Microparticles with or without Adjuvant MP

#### 3.3.1. Adjuvanted Gonococci Vaccine Microparticles Enhanced Nitric Oxide Release from Induced Macrophages

**Figure 2:**
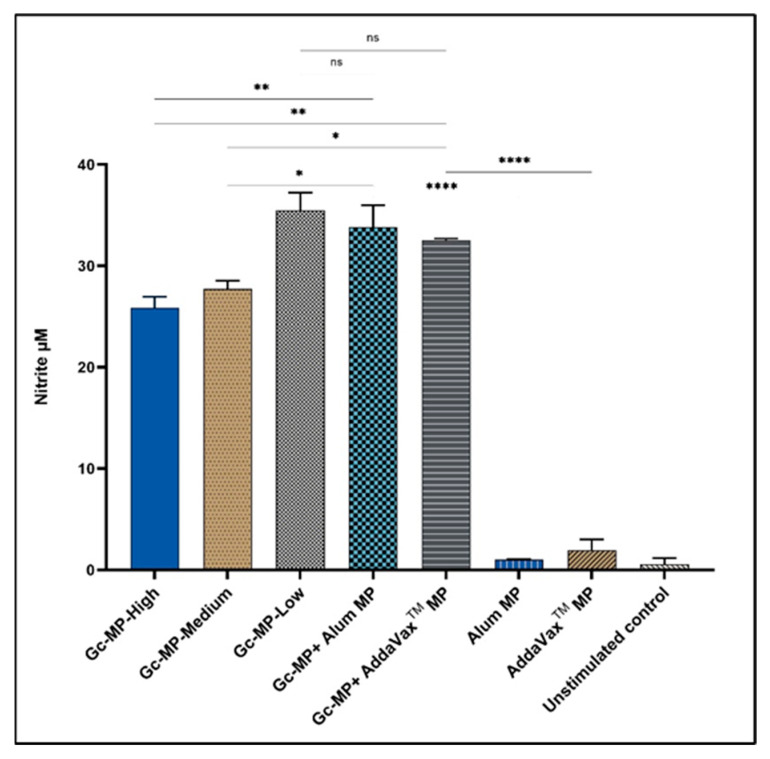
Nitric oxide release from GFP-LC3-RAW264.7 macrophages induced with gonococci vaccine microparticles (Gc-MP) with or without adjuvant MP. Macrophages were adjusted to 1 × 10^6^ cells/mL and induced with various doses of vaccine microparticles for 18 h. Gonorrhea vaccine microparticles Gc-MP-High 100 µg/mL, Gc-MP-Medium 50 µg/mL, Gc-MP-Low 25 µg/mL, Gc-MP with Alum microparticles (Gc-MP+ Alum MP) 50 µg/mL of each, Gc-MP with AddaVax™ microparticles (Gc-MP+ AddaVax™ MP) 50 µg/mL of each. Unstimulated cells, Alum MP, and AddaVax™ MP alone were used as controls. Nitrite accumulation in supernatants was measured using the Griess reaction. All the experiments were repeated three times. Data is expressed as mean ± SD, Ordinary one-way ANOVA test, post-hoc Tukey’s multiple comparison test. ns, no significant, * *p* < 0.05 significant, ** *p* < 0.01 very significant, **** *p* < 0.001 extremely significant.

The potential of the adjuvanted gonococci vaccine microparticles (Gc-MP+ Alum MP or AddaVax™ MP) to induce an immune response was assessed by measuring the amount of nitric oxide released by RAW264.7 macrophages. The released nitric oxide is very unstable as a free radical and converts to the stable nitrite form that is measured in supernatants using the Griess reaction [40]. The results show that adjuvanted gonococci vaccine MP (Gc-MP+ Alum MP and Gc-MP+ AddaVax™ MP) induced significantly higher amounts of nitric oxide release compared to macrophages exposed to Gc-MP alone (Figure 2). Interestingly, adjuvants alone (Alum MP and AddaVax™ MP) did not induce nitric oxide release similar to unstimulated control macrophages (Figure 2).

#### 3.3.2. Cytotoxicity Evaluation of Gonococci Vaccine MP

**Figure 3:**
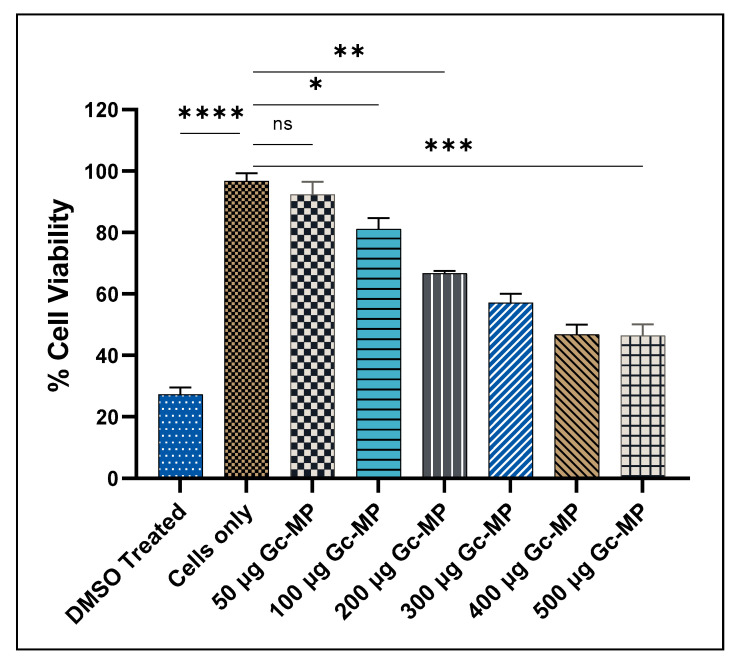
Dendritic cell (DC 2.4) viability after exposure to different amounts of gonococci vaccine microparticles measured using MTT assay. DC 2.4 cells (2.5 × 10^4^ cells/well) were exposed to increasing doses of Gc-MP (50 to 500 µg/well) or DMSO (50 µL) and incubated at 37 °C for 48 h. All the experiments were repeated as three independent experiments with three replicates in each experiment. Data are expressed as mean ± SD, One-way ANOVA- Brown-Forsythe and Welch ANOVA test. ns, no significant, * *p* < 0.05 significant, ** *p* < 0.01 very significant, *** *p* < 0.05 extremely significant, **** *p* < 0.001 extremely significant. Dimethyl sulfoxide (DMSO); Cells only: Unstimulated DC 2.4 were used as the control.

Here we assessed the cytotoxicity of Gc-MP increasing doses in dendritic cells (DC 2.4) using the MTT (3-(4,5-Dimethylthiazol-2-yl)-2,5,diphenyltetrazolium bromide) assay [43]. Cytotoxicity assay showed that within the tested concentration ranges (50 to 500 µg), Gc-MP were non-cytotoxic towards the DCs, the cells were 50–80% viable after 48 h of exposure when compared to the control DMSO (Figure 3). However, the percentage of viable DCs were reduced in a dose-dependent manner when compared to unstimulated DCs exposed only to DMEM medium without any MP (Figure 3). The data suggests that the lower doses of Gc-MP were not cytotoxic after 48 h of exposure. Therefore, 100 µg dose of Gc-MP formulation was selected for further studies.

#### 3.3.3. Dendritic Cell Uptake of Microparticles

**Figure 4:**
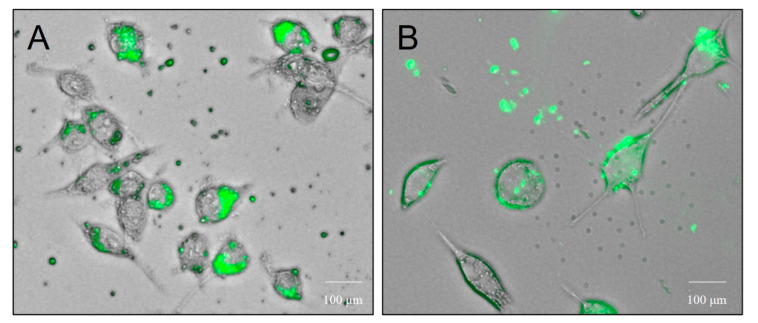
Dendritic cell (DC) uptake microparticles. Representative images of the uptake of microparticles by antigen presenting dendritic cells observed using the fluorescence microscope (**A**,**B**). The FITC-BSA microparticles were rapidly taken up by the dendritic cells and were accumulated in the cytosol and the vacuolar compartments (40×).

The uptake of microparticles by antigen presenting dendritic cells was observed using fluorescence microscope. FITC-labelled microparticles were rapidly taken up by dendritic cells and were accumulated in the cytosol and the vacuolar compartments (Figure 4A,B).

#### 3.3.4. Expression of Antigen Presenting and Co-Stimulatory Molecules on the Surface of Dendritic Cells

**Figure 5:**
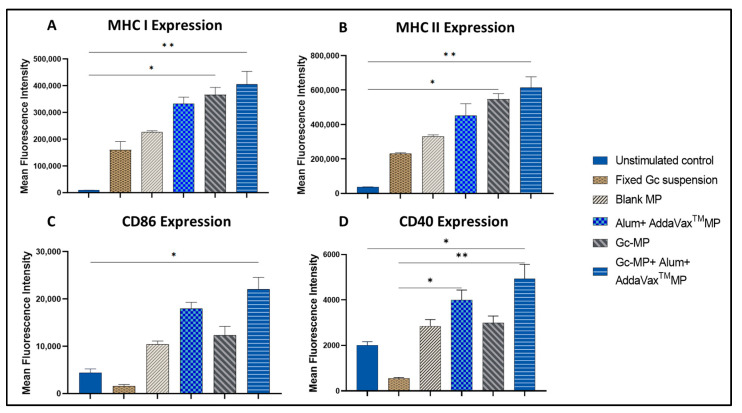
Expression of MHCI, MHCII, and co-stimulatory molecules CD40, CD86 on the surface of the dendritic cells induced with adjuvanted gonococci vaccine microparticles. DC 2.4 cells (5 × 10^4^ cells per well) were induced with Gc-MP (100 µg/well) alone or combined with adjuvants (Alum MP or AddaVax™ MP) or inactivated or fixed whole-cell gonococci in suspension (100 µg/well) then incubated at 37 °C for 24 h. The surface expression of MHCI (**A**), MHCII (**B**), CD86 (**C**) and CD40 (**D**) on the DCs induced by adjuvated Gc-MP was compared to unstimulated DC 2.4 control cells. All data were repeated as three independent experiments with three replicates in each experiment. Data are expressed as mean ± SD, One-way ANOVA-Kruskal-Wallis test. * *p* < 0.05 significant, ** *p* < 0.01 very significant.

The data show that surface expression of MHCI and MHCII were significantly higher (*p* < 0.01) in dendritic cells exposed to adjuvanted vaccine MP compared to whole-cell inactivated gonococci in suspension without microparticles (Figure 5A,B). Similarly, the expression of co-stimulatory molecules CD40 and CD86 on the DCs was also significantly higher in the adjuvanted vaccine group as compared to untreated control (*p* < 0.05) (Figure 5C,D). The untreated DC 2.4 control group did not induce the expression of co-stimulatory molecules. The empty MP did not induce any significant response (data not shown) as previously reported [32].

### 3.4. Expression of CD95 Death Receptor on the Surface of Antigen Presenting Cells Induced with Gonococci Vaccine Microparticles

**Figure 6:**
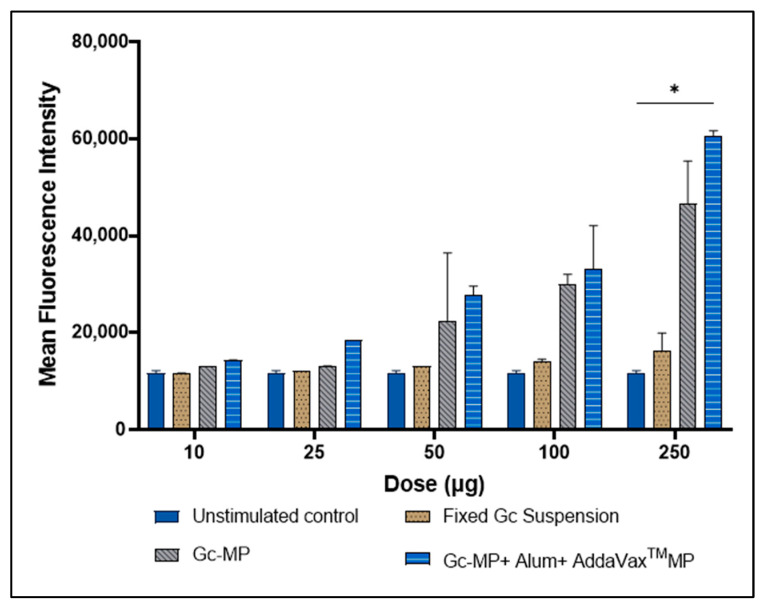
Induction of the surface expression of CD95 on dendritic cells induced with different treatments. DC 2.4 cells (5 × 10^4^ cells per well) were exposed to increasing doses of Gc-MP (10, 25, 50, 100 and 250 µg/well) alone or combined with adjuvants (Alum MP or AddaVax™ MP), or inactivated or fixed whole-cell gonococci in suspension (100 µg/well), then incubated at 37 °C for 24 h. The expression of CD95 receptor was measured using flow cytometry. All data points were repeated as two independent experiments with three replicates in each experiment. Data are expressed as mean ± SD, Two-way ANOVA, post-hoc Dunnett’s multiple comparison test. * *p* < 0.05 significant.

We investigated whether whole-cell formalin-fixed *N. gonorrhoeae* in suspension or formulated in MP can induce the expression of CD95 death receptor on the surface of APC in vitro. Murine DCs were exposed to gonococci vaccine MP in a dose-dependent manner. The viability of DCs was assessed using the trypan blue exclusion assay. The expression of CD95 increased as the concentration of MP was increased from 10, 25, 50, 100 and 250 μg/well in DCs. The data suggested that lower doses did not have significant CD95 expression; however, an increased gonococci vaccine MP dose above 100 μg increased the expression of CD95. Higher CD95 expression may lead to the death of APCs, and consequently sub-optimal immune response to the vaccine (Figure 6).

## 4. Discussion

**Figure 7:**
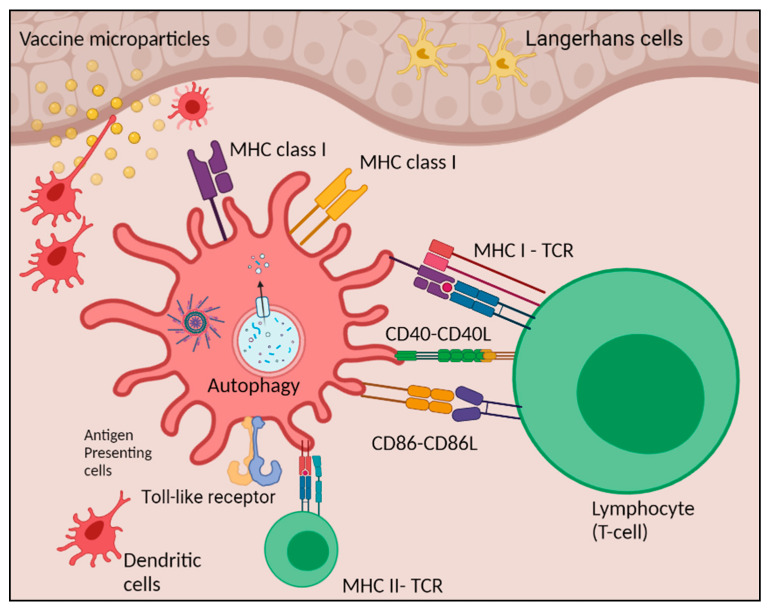
Schematic presentation of immune cells activation post vaccine MP uptake. Adjuvanted gonococci microparticles (Gc-MP+ Alum MP+ AddaVax™ MP) are effectively taken up by the antigen presenting cells (APCs), processed in the cytoplasm of the APCs, induced autophagosomes, and presented to the antigen presenting molecules (MHC I and II) with the help of co-stimulatory molecules (CD86 and CD40).

We assessed the novel adjuvanted gonococci vaccine microparticle formulation for optimal antigen presentation. The process of antigen presentation by APCs represents an incredible endeavor of evolution to provide and guarantee maximal efficiency and specificity of an adaptive immune response. We found that the adjuvanted Gc-MP+ Alum MP+ AddaVax™ MP are effectively taken up by the APCs, processed, induced autophagosomes, and enhanced the surface expression of the antigen presenting molecules (MHCI and MHCII) and co-stimulatory molecules (CD86 and CD40). This antigen presentation will facilitate effective stimulation of naive T-helper lymphocytic cells leading to the generation of specific adaptive immune responses (Figure 7) [44].

The microparticulate vaccine platform offers several advantages over conventional vaccines as MP slowly releases antigens from the polymer matrix which aid in antigen presentation by recruiting molecules of the innate immune system [45]. MP vaccine formulation enhances the stability of the associated antigens [46] and enables concomitant delivery of the vaccine antigen and adjuvant [46]. The microparticulate vaccine is a dry formulation and thus thermostable [47] i.e., it does not require the cold chain, which facilitates ease of transportation and administration. We used a biodegradable pre-cross-linked albumin matrix to form the gonococci vaccine MP [35]. The albumin-based polymer matrix enables sustained release of the antigen and mimics the chemical conjugation process to a protein carrier, thereby help in binding to the albumin receptors [48]. We formulated MP containing Alum (Alhydrogel^®^) and AddaVax™ (Analog of MF59) as vaccine adjuvants. Alum induces Th-2 mediated immune response by releasing the reactive oxygen species and activating the NLRP3 inflammasome in the APCs, whereas the adjuvant MF59^®^ is known to recruit sentinel cells at the site of administration, thereby creating an immunocompetent environment [49,50,51,52]. The addition of adjuvants into the particulate vaccine can heighten immunogenicity by significantly enhancing antigen presentation and overcoming the inhibitory effects of traditionally formulated vaccine antigens.

APCs are an essential component of immunity against any infectious diseases. These professional cells activate the naïve T cells, which drive the protective immunity against infection. As previously demonstrated by scanning electron microscopy, the formalin-fixed whole-cell *N. gonorrhoeae* bacteria were found to be in their native form and intact (not lysed) before processing into microparticulate vaccine [30]. Here, we demonstrated that DCs and macrophages, upon stimulation with gonococci vaccine MP, released nitric oxide indicating the immune stimulatory activity of this vaccine formulation. The adjuvanted Gc-MP were recognized by the APCs and were able to stimulate the release of nitric oxide, an essential vital immunogenicity marker [31,53]. Nitric oxide is an innate immunity marker released by APC upon stimulation by an antigen measured using Griess assay.

The desired vaccine formulation must be safe and non-cytotoxic to the APC-like dendritic cells (DCs). Suboptimal antigen presentation may result from cytotoxic effects of vaccine formulation components on APCs. Lower doses of Gc-MP were not cytotoxic after 48 h of exposure.

Autophagy is a homeostatic process that has been implicated in various physiological and pathological processes regulating immune responses, autoimmunity, cancer, infection, and neurological disorders. It plays a vital role in host defense by eliminating invading pathogens and facilitating antigen presentation, thereby contributing to immunity [38]. Recent studies have discussed the process of autophagy in APCs and its context in the development of immunity [54,55]. Autophagy involves antigen processing and presentation in the DCs or macrophages for the MHCI and MHCII. We showed the increased formation of autophagic vacuoles in the APCs upon exposure to gonococci vaccine MP, and the presence of adjuvants further enhanced autophagy induction which is critical for enhancing antigen presentation.

Autophagy and apoptosis are both catabolic processes in the cells important for maintaining homeostasis. Literature suggests crosstalk between apoptosis and autophagy [56]. Caspases are involved in apoptotic cascades and, when activated, cleave the autophagic proteins such as Atg3, Atg4D, Atg5, and Atg7, inactivating their autophagic pathway function. Various cancer studies involve transforming the caspase cleaved autophagic proteins into apoptotic proteins to induce cell death. Moreover, autophagy can modulate the caspases and affect apoptosis [57]. CD95 is a membrane protein commonly known as the death receptor. The binding of the CD95-ligand to CD95 leads to activation of the death pathway and is orchestrated by the action of caspases [58,59]. Caspase-8 is an important trigger involved in death-receptor-induced apoptosis [60]. This caspase also has an important role in the regulation of autophagy. At the time of death-receptor-induced apoptosis, caspase-8 cleaves Atg3 autophagic protein and inactivates the autophagic pathway preventing T-cell activation [61]. It has been shown that some bacterial capsular polysaccharides such as *Cryptococcus neoformans,* glucuronoxylomannan induce upregulation of CD95-ligand on APCs and, thereby, apoptosis of the T cells [62,63]. This phenomenon was reported for a human protozoan parasite *Trypanosoma cruzi* [64]. Upon immunization with a recombinant adenovirus expressing the parasite antigen, there was an induction of apoptosis by CD95-ligand overexpression, leading to sub-optimal CD8+ T cell response and failure of the vaccine strategy [65]. We have previously reported that a high dose of meningococcal serogroup A CPS as vaccine antigen induced the expression of the death receptor CD95 in murine DCs as well as macrophages leading to cell death [31]. Currently, it is not known if *N. gonorrhoeae* can induce CD95, which may dampen adaptive immune responses. We examined whether gonococci may be inducing the death receptor pathway, leading to death of APCs. Our data show a dose-dependent induction of CD95 in APCs induced with adjuvanted gonococci MP. Overexpression of the CD95 has been attributed to cell death as it binds to CD95L, the death receptor [58]. A high dose of the vaccine MP greater than 100 µg led to decreased cell viability of the APCs tested in vitro. Further experiments are warranted to examine in vivo the induction of CD95 on APCs in mice vaccinated with adjuvanted gonococci MP.

## 5. Conclusions

We successfully formulated an inactivated whole-cell gonococci vaccine in a microparticle form adjuvanted with Alum or AddaVax™. The slow release of antigens and adjuvants from microparticle vaccine formulation is a key for enhanced immune responses. In vitro analysis of this formulation suggests a potential vaccine candidate that retained immunostimulatory activity while being non-cytotoxic. The adjuvanted gonococci vaccine MP induced autophagy, which enhances antigen presentation. This adjuvanted gonococci vaccine formulation induced significantly higher expression of co-stimulatory molecules CD40, CD86, and antigen presenting molecules MHCI, MHCII expression in APCs. Ongoing experiments are currently testing the immunogenicity of this adjuvanted gonococci vaccine formulation in vivo using mouse model.

## Figures and Tables

**Table 1:** Characterization of gonococci vaccine and adjuvant microparticles.

	Gc-MP	Alum MP	AddaVax MP
**Protein content in fixed Gc**	2.5 mg/mL	-	-
**Percent yield**	85% *w*/*w*	88% *w*/*w*	84% *w*/*w*
**Particle size**	3.5 ± 1.2 μm	4.3 ± 0.7 μm	2.18 ± 0.9 μm
**Polydispersity index (PDI)**	0.34	0.60	0.558
**Zeta potential**	−25 ± 5.79 mV	−22 ± 2.33 mV	−16.95 ± 1.53 mV

## Data Availability

Not applicable.
